# Comparisons of Trunk Motions and Low Back Injury Risk between Alternative Hotel Room Cleaning Methods

**DOI:** 10.3390/ijerph192214907

**Published:** 2022-11-12

**Authors:** W. Gary Allread, Pamela Vossenas

**Affiliations:** 1 SRI-Ergonomics, The Ohio State University, Columbus, OH 43210, USA; 2 Department of Labor Studies, CUNY School of Labor and Urban Studies, New York, NY 10036, USA

**Keywords:** hotel room cleaners, housekeeping, low back disorder risk, adjustable, long-handled cleaning tools, ergonomic interventions, work-related musculoskeletal disorders

## Abstract

Hotel room cleaners frequently report job-related pain, with high rates of work-related musculoskeletal disorder injuries established for this group of workers. Surprisingly, there is limited published research documenting the impact of interventions to reduce ergonomic-related injury risks specific to hotel room cleaners’ job tasks. In this study focused on hotel bathroom-cleaning and furniture-dusting tasks, twelve experienced hotel room cleaners used their standard method and a risk-reduction method—a tool with a handle that could extend, to perform these tasks. The female study participants’ average age was 45.3 (*SD* 8.7) years with an average of 10 years of work experience as cleaners (range: 0.8–26.0 years). Trunk kinematics and a low back injury risk assessment were measured using the Lumbar Motion Monitor. All study metrics were significantly reduced when cleaning tasks involved use of adjustable, long-handled tools (*p* < 0.05). This study demonstrated that commonly available cleaning and dusting tools with extendable handles can significantly reduce low back injury risk among hotel room cleaners and potentially reduce injury risk to other body parts known to be the site of musculoskeletal disorders in this workforce. The study findings suggest that cleaning or housekeeping jobs in other industries where these same tasks are performed could benefit from use of extended-handle tools like those investigated here.

## 1. Introduction

Approximately 720,000 individuals are employed as ‘Maids and Housekeeping Cleaners’ in the United States in a variety of industries [1]. A predominantly (89%) female workforce [2], the ‘Traveler Accommodation’ industry is the leading employer (42.6%) of this occupation classification [3]. Within the hotel industry, maids and housekeeping cleaners account for the largest proportion of all employees (22%), making the *hotel room cleaner* occupation a hotel’s largest workforce [3].

Hotel room cleaners perform a wide variety of cleaning tasks, including making beds, dusting and polishing furniture, vacuuming carpets, washing windows and walls, cleaning bathroom fixtures, and moving furniture within the guest room [4,5,6]. In a hotel, room cleaners are a job classification found within the housekeeping department. The number of housekeeping job tasks required of these individuals has increased, as hotels have gradually added more room amenities for its guests [7].

Hotel room cleaners often report symptoms of musculoskeletal disorders (MSDs). In a survey of nearly 950 room cleaners across five different hotels [8], 75% reported having pain or discomfort within the past year and believed it to be caused by or made worse by the work. Further, 94% stated that the pain began after beginning the hotel room cleaning job. The prevalence of reported low back pain among a sample of hotel room cleaners was found to be over 58% [9]. In another study, female cleaners reported musculoskeletal pain or discomfort in their necks, shoulders, elbows, hands/wrists, low backs, hips, knees, and ankles at similar or higher rates than their counterparts working in other occupations [10]. Finally, in Burgel et al.’s survey of nearly 500 hotel room cleaners [11], more than half reported experiencing “severe” or “very severe” shoulder pain.

In addition to the MSD symptoms claimed by hotel room cleaners, high rates of injury have been reported among this workforce as well. A review by Buchanan et al. [12] of OSHA 300 injury logs over a three-year period from hotels in five major U.S. cities found MSD incidence rates to be higher for room cleaners than for any other hotel services job category studied (i.e., banquet servers, stewards/dishwashers, cooks/kitchen workers, other jobs). Most MSDs were to the back, followed by the hands/wrists and shoulders. Hotel room cleaners also had the second highest rate of acute trauma. These findings are comparable to national injury data by industry. Hotel and motel employees were found to have a higher overall injury rate (2.9 non-fatal incidences per 100 full-time workers) than those working in manufacturing (2.6), the service industry as a whole (2.1), and across all private industry (2.2) [13].

Yet, for the hotel industry, there is limited scientific literature about interventions to improve worker health outcomes overall, including injury prevention [14]. A focus group study of hotel room cleaners identified ergonomics as an intervention need [15]. Research about ergonomic interventions for hotel housekeeping tasks involving hotel room cleaners as study participants are limited to certain cleaning tasks. For bedmaking tasks, study authors found that the use of a mattress lift tool and fitted sheets reduced the number of times a mattress was lifted and decreased physical exertion in the back and upper extremities, trunk movement and forearm muscle activity [16]. For the task of pushing a housekeeping cart, a significant decrease in pull weights was found at both a full stop and while moving by changing the type of ball bearings and the material that the wheels were made of [17]. Both of these studies demonstrate the success of applying ergonomic interventions to hotel room cleaning tasks.

Several studies performed in a non-hotel setting have shown that particular cleaning tasks can be made easier by using cleaning tools with different or modified handles, resulting in improved biomechanics of the job tasks and worker interaction with the equipment. When using a long-handled tool compared to a rag for cleaning surfaces, Liu et al. [18] found that study subjects expended a lower 50th percentile force at all task heights and had high preference rates when using the tool at high heights. Kumar et al. [19] used professional cleaners to compare two floor-cleaning tools—one having a conventional, straight handle, and one with a redesigned, curved handle—as these individuals performed their regular job tasks. The metrics oxygen consumption, heart rate, and trunk posture were found to be significantly lower when the modified tool was used. Improved trunk flexion angles allowed cleaning to be performed in more upright postures. In addition, worker interaction with the study cleaning tool resulted in lower subjective ratings of perceived effort during the study task. Janowitz et al. [20] compared use of a handled tool to clean a bathtub/shower area with that of a sponge and spray cleaner. They found that trunk, wrist, and shoulder angles were significantly less deviated when using the handled tool. Subjectively, cleaners reported a preference for this tool and found it more comfortable and easier to use than the sponge. Although not a focus of this study, it also was found that study subjects had improved footing using the tool, as more time was spent with feet on the bathroom floor instead of inside the tub. Kumar et al. [21] studied experienced cleaners who mopped staircases using either a standard (non-adjustable) or adjustable mop stick handle. Shoulder muscle activity in the right/dominant side of subjects was found to be significantly lower when the adjustable handle was used. Finally, Wallius and colleagues [22] reviewed several floor mopping studies and categorized the findings by level of evidence to support interventions to reduce job task physical exposure. The authors concluded there to be “moderate evidence” that mop handle designs can have a positive impact on reducing such exposures and are “a practice to be considered”.

These intervention studies, though not performed in a hotel setting, have demonstrated the positive benefits of performing cleaning tasks using modified tools or work practices. However, most have reported on whole-body or upper extremity outcomes rather than the individual body part where many hotel room cleaners report pain or become injured—the back.

In a timed study of hotel housekeeping tasks, cleaning the bathroom and furniture cleaning were among the top five individual room cleaning tasks when accounting for time spent cleaning [23], with combined bathroom-cleaning tasks accounting for 33% of room cleaning time and cleaning furniture for 14%. When studying cardiovascular demands for hotel room cleaning work, dusting and bathroom-cleaning ranked second and third, respectively, for peak percent heart rate reserve among five room cleaning tasks studied [24]. Thus, ergonomic interventions need to be prioritized for these two key hotel room cleaning tasks.

This study was conducted for three primary purposes: (1) to measure how room cleaners must move their trunks to perform bathroom-cleaning and furniture-dusting tasks in hotel guest rooms; (2) to determine if alternative work methods or the use of alternative cleaning tools alters hotel room cleaners’ trunk motions and, subsequently, their risk of developing a low back disorder; and (3) to contribute towards filling the gap in the literature on injury risk-reduction methods for bathroom-cleaning and furniture-dusting tasks performed by hotel room cleaners.

## 2. Materials and Methods

### 2.1. Approach

In this study, three-dimensional spine motions and subsequent Low Back Disorder (LBD) risk estimates were measured as hotel room cleaners performed a variety of common room cleaning tasks using various work methods. From this, determinations were made as to whether use of these alternative work methods were effective at reducing trunk motions and LBD risk and, therefore, should be considered as tools for improved work practices for hotel room cleaning tasks.

### 2.2. Participants

A total of twelve English- or Spanish-speaking female hotel room cleaners participated in this study. This sample size, determined using Pass software (NCSS, Kaysville, UT, USA), was found to be sufficient to detect a moderate effect size in trunk kinematic variables with a power of 0.80 and significance level alpha = 0.05, prior to the start of the study. It also helps to account for variations across individual differences, such as age, body size, and work experience. These employees had worked as room cleaners in the study hotel an average of 6.4 years (range: 0.8–10.0 years) and, as a career, had an average of 10.5 years of experience as cleaners (range: 0.8–26.0 years). All worked at the same unionized hotel located in Southern California. Their average (standard deviation) age and standing height (verbally reported) were 45.3 (8.7) years and 159.5 (5.6) cm, respectively. Potential subjects were excluded from participating if they had any history of an LBD or if they were experiencing physical pain or discomfort at the time of the study.

### 2.3. Study Design

**Figure 1:**
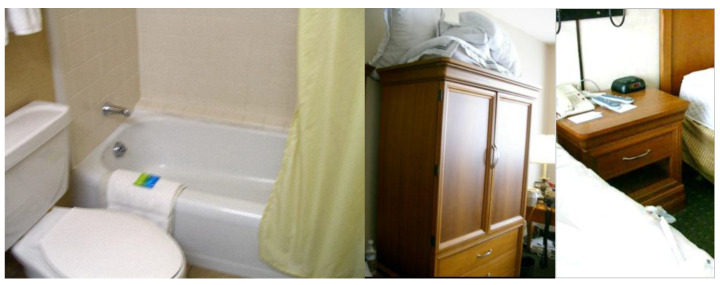
Room layout used for study data collection.

The study occurred within the actual hotel where the subjects were employed, so they could perform the cleaning tasks of interest in an environment where they were comfortable and familiar with the room layout and its contents. The guest room (Figure 1) was typical of those found in mid-priced hotels for a standard room.

**Figure 2:**
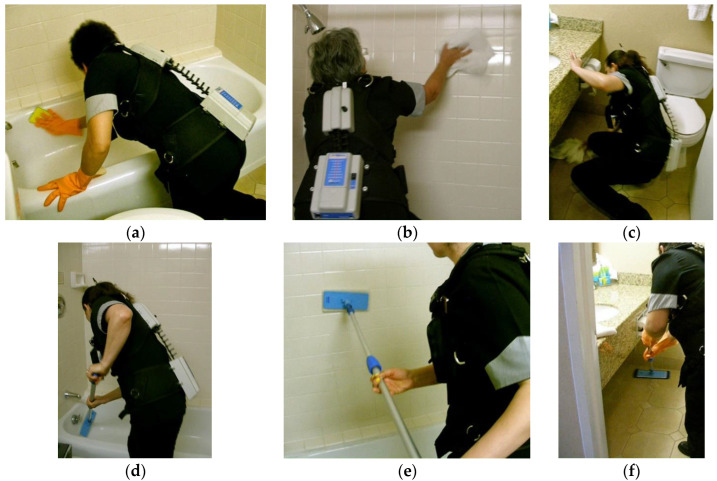
Bathroom-cleaning tasks studied. Wiping down the tub, shower wall tile, and floor using standard methods are shown in (**a**–**c**), while those tasks done using a tool with an adjustable, long-handle are shown in (**d**–**f**).

**Figure 3:**
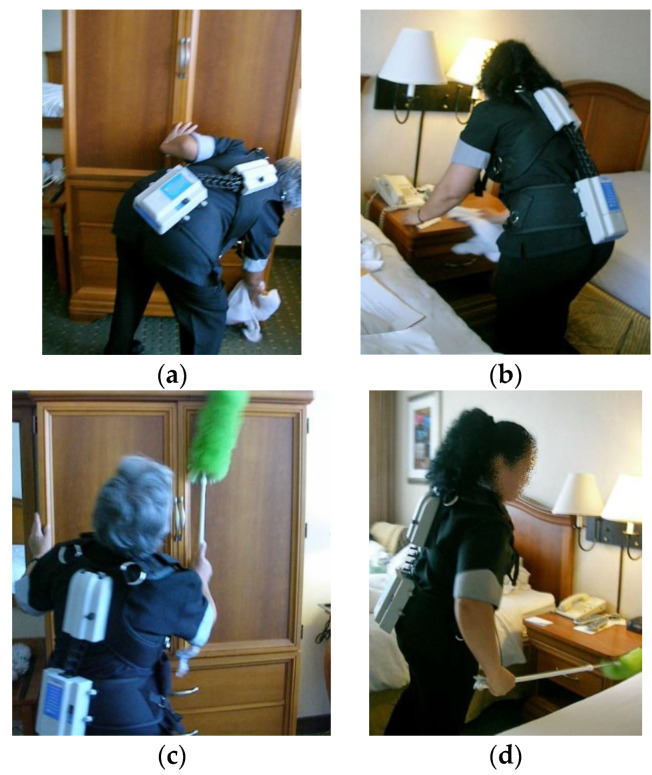
Dusting tasks studied. Dusting an armoire and nightstand using standard methods are shown in (**a**,**b**); those tasks done using a long-handled, extendable tool are shown in (**c**,**d**).

There were ten independent variables studied, which were grouped into two cleaning functions: bathroom-cleaning (six) and dusting (four). The bathroom tasks studied (Figure 2) involved use of a rag to clean the bathtub surface, the shower walls, and the floor, and then these same tasks done with an adjustable, long-handled tool onto which the cleaning rag was placed. The dusting tasks involved cleaning the surface of the room’s armoire and nightstand using a rag and again with a microfiber duster with an extendable, long handle (Figure 3). A repeated measures experimental design was used for both tasks, in which each subject performed each cleaning condition. Within each cleaning scenario, the sequence of tool type was randomized. The order in which each cleaning task was performed also was randomized.

The dependent variables included trunk kinematic measures (i.e., positions, velocities, and accelerations of the lumbar spine in its three cardinal planes of motion—lateral (side-to-side), sagittal (flexion and extension) and transverse (twisting)) and an assessment of the job task’s risk of creating high rates of LBDs among those who perform the job.

### 2.4. Tools

Two types of adjustable, long-handled tools were used. For bathroom-cleaning tasks, the adjustable tool (Figure 2d–f) was constructed of aluminum, weighed approximately 0.7 kg, and could extend from 61 to 122 cm. The adjustable tool was manufactured by Ecolab™, #96212288 and is sold as part of its Housekeeping Tool Kit. The duster (Figure 3c,d) had a metal handle that could extend to 130 cm; it weighed about 0.2 kg. It is similar to dusters manufactured by Sabco™ #SAB42022. These tools are commonly available.

### 2.5. Data Collection

An Acupath lumbar motion monitor (LMM) was used to measure the low back motions of subjects as they performed the study tasks. The LMM (shown in Figure 2 and Figure 3 being worn by room cleaners) is a tri-axial electro-goniometer that measures position of trunk movement in the three planes of the body. Instantaneous data recorded from the LMM were transmitted via digital telemetry to a laptop computer, where custom software processed the data to calculate trunk velocities and accelerations. Researchers from around the world have used the LMM to measure a wide variety of materials handling tasks and other physical activities that involve use of the trunk [25,26,27,28,29]. Its ability to accurately and reliably measure lumbar spine kinematics has been previously validated [30] and tested [31].

### 2.6. Procedure

All data were collected in a standard guest room reserved at the hotel that employed the individuals who participated in the study. Hotel room cleaners had been informed of the study volunteer opportunity through their union. Upon arrival, the cleaner provided written consent to be monitored, as required by the university’s institutional review board. The subject was then given a brief explanation on the cleaning tasks to be performed and shown the tools that were to be used to perform these activities. Instructions were provided in English or Spanish based on the worker’s preference. For bath-cleaning tasks, water was splashed into the tub and over shower walls, and subjects were instructed to wipe down the entire surface areas as if the area was being cleaned for a guest. Subjects also were instructed to wipe across the entire floor surface during these cleaning conditions. For dusting tasks, subjects were asked to wipe across all exposed furniture surfaces. Subjects were instructed to perform all tasks at a pace comparable to that required of them during a normal workday. The area of total bathroom surfaces cleaned is estimated at 12 m^2^ and 6 m^2^ for wood surfaces dusted.

In most cases, subjects were highly experienced room cleaners (see Section 2.2) and reported having previously used the same or similar tools for other job tasks during their careers. Regardless, subjects were encouraged to adjust the tools to their desired lengths and practice using them before data collection began. As a result, it was observed that these room cleaners performed their task functions in much the same manner. Finally, the subject was shown the LMM and given a brief explanation of its purpose. After any questions about the testing procedure or tools were answered, the monitor was placed on the subject, and data-gathering began.

Subjects performed the bathroom-cleaning or dusting test conditions together; however, the order in which each group of tasks were done was randomized, as was the order of each condition within the set of cleaning areas. Subjects performed each complete task condition a minimum of three times. This number of task trials was selected, as it had been previously found that collecting three repetitions of a job task was sufficient to capture the variability of an occupational activity [32]. To prevent the possibility of fatigue, subjects were encouraged to take as many rest breaks as they deemed necessary. The LMM was removed from the subject once all data were gathered.

### 2.7. Data Analysis

Signals recorded from the LMM were stored on a laptop computer. The customized data collection software was also used to process these signals and calculate position, velocity, and acceleration in the trunk’s lateral, sagittal, and transverse (twisting) planes of motion. For each subject, trunk kinematic data were averaged across each set of unique room-cleaning tasks gathered.

The customized data collection software (Ballet 2.0) also assessed the probability that the tasks studied were like jobs that previously reported high rates of LBDs. This previously developed model (“LBD risk”) was based on workplace and lumbar motion data collected on adults in over 400 jobs across diverse industrial facilities [33]. Statistical analyses of these data found that a combination of five specific workplace and trunk kinematic variables best distinguished the “high risk” group (defined as jobs having twelve or more LBDs annually per 100 full-time employees) from the “low risk” group (jobs having zero LBDs and no job turnover). Two of the five factors were workplace variables—the job’s hourly item lifting frequency and the maximum external moment generated about the spine (i.e., the product of the load weight and the distance it is held from the lumbo-sacral joint) as the item was handled. The remaining three model factors were trunk kinematic variables taken from the LMM—the maximum spinal lateral velocity, the maximum spinal sagittal flexion, and the average spinal twisting velocity produced during the task. A risk value generated for a task using these five factors (on a scale from 0 to 100%) represents the probability that the task observed generally fits the profile of high-injury jobs previously studied. This risk model was validated [34].

Student’s *t*-tests of paired samples, comparing methods for the various room-cleaning tasks (i.e., rag or sponge vs. sponge on adjustable, long-handled tool; rag vs. adjustable, long-handled duster) were performed using Statistica (StatSoft, Tulsa, OK, USA), to determine if there were differences in trunk motions and LBD risk. Results with a *p* value ≤ 0.05 were considered statistically significant.

## 3. Results

Means and standard deviations for the dependent measures are shown in Table 1 and Table 2 for bathroom-cleaning and dusting tasks, respectively. These tables also note which conditions produced outcomes that were statistically significant from one another. Note that, although LMM data analyses generate numerous trunk kinematic values (e.g., left lateral position angle, sagittal extension angle, counterclockwise trunk rotation angle), only those believed to be of most biomechanical relevance to this project are presented.

### 3.1. Bathroom-Cleaning Tasks

As shown in Table 1, all trunk kinematic measures were significantly lower when the tasks were done using an adjustable, long-handled tool with a rag or sponge attached, compared to using the standard cleaning method (i.e., a rag held in the hand). In other words, regardless if subjects were wiping down the bathtub, shower walls, or floor, their trunk positions, on average, were less deviated, and they moved their backs more slowly, when using the tool. This was found for all side-bending (lateral), forward-bending (sagittal), and trunk-twisting (transverse) back motions. For example, the three bathroom tasks performed using standard wiping methods resulted in average spine flexions ranging from 14 to nearly 27 deg; however, use of the tool allowed these tasks to be performed while standing nearly upright (i.e., from 2 deg of extension to 7 deg of flexion). In addition, the computed LBD risk values, also shown in Table 1, were significantly lower when the adjustable, long-handled tools were used for these three bathroom-cleaning tasks.

### 3.2. Dusting Tasks

For furniture-dusting, most trunk motions significantly decreased when performed using an extendable, long-handled tool, compared to a rag. As shown in Table 2, all lateral and sagittal plane trunk motions were lower during tool use, regardless of the piece of furniture (armoire or nightstand) being cleaned. Standard dusting methods produced an average of about 24 deg of sagittal flexion, while dusting with the tool resulted in flexions of 11 deg or less. However, there were no differences found in the amount of transfer plane (twisting) motions produced when performing the tasks between dusting methods or across furniture pieces. Similar to the bathroom-cleaning findings, the LBD risk values were significantly lower when the dusting tasks were performed using a duster with an extendable, long handle, compared to a rag.

## 4. Discussion

### 4.1. Bathroom-Cleaning and Dusting Tasks

**Figure 4:**
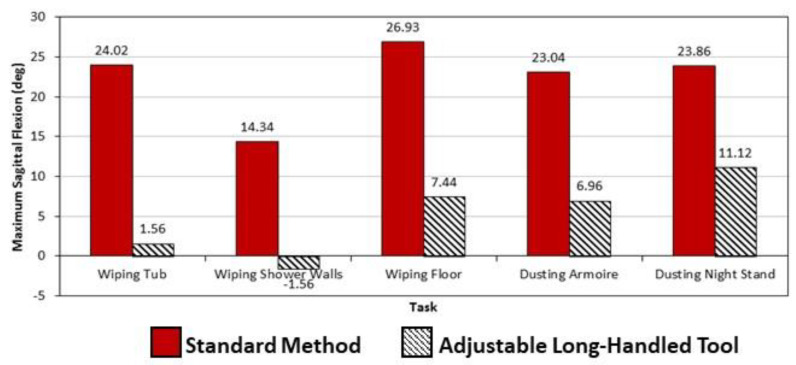
Differences in average sagittal flexion angles between cleaning methods of five bathroom and furniture cleaning methods. All were significantly different (*p* < 0.05).

This study has demonstrated that adjustable, long-handled tools can greatly reduce the trunk kinematics required of hotel room cleaners as they carry out several commonly performed cleaning tasks, such as bathroom-cleaning and furniture-dusting. In nearly all cases, tools with extendable, long handles used to wipe down bathroom tubs, shower walls, and floors and to dust furniture not only reduced three-dimensional trunk deviations and the speed with which the trunk moved to perform the tasks but also lowered the low back injury risk level of the task. In addition to these differences being statistically significant, they also demonstrate the practical improvement to be derived from use of these modified work practices. Additionally, average sagittal flexion angles were significantly lower for all bathroom and furniture cleaning tasks using the study tools. As shown in Figure 4, subjects, on average, cleaned bathrooms and dusted furniture with the tool using nearly upright or slightly flexed sagittal positions of no more than about 11 degrees. However, the standard method required forward flexion angles averaging approximately 23 degrees or more for four out of the five tasks studied (the only exception was wiping shower walls, which had an average flexion angle of about 14 degrees). Other researchers have noted these types of flexion discrepancies to be related to the development of spine-related disorders. In a study of automobile assembly workers [35], Punnett et al. found that, compared to those whose jobs allowed them to work while nearly upright (less than 20 deg of forward flexion), those who worked in mildly flexed forward postures were nearly five times more likely to report or exhibit symptoms of a back-related disorder. This may help to at least partially explain why hotel room cleaners report more back-related MSDs than to any other individual body part [12].

**Figure 5:**
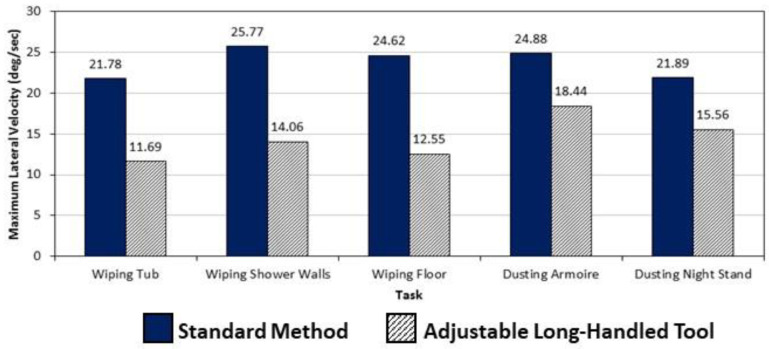
Differences in average maximum lateral trunk velocities between cleaning methods of five bathroom and furniture cleaning methods. All were significantly different (*p* < 0.05).

**Figure 6:**
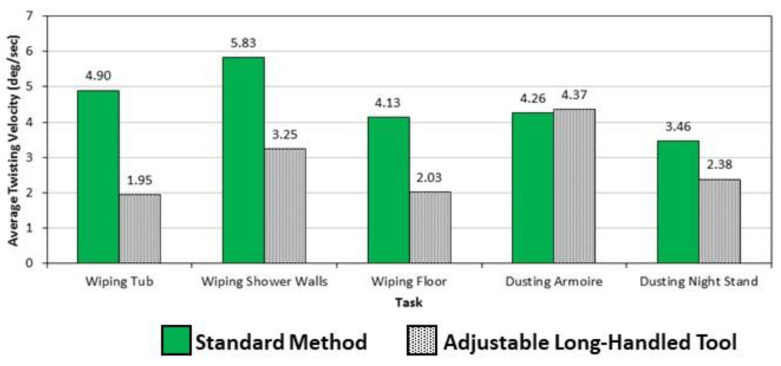
Differences in average transverse trunk velocities between cleaning methods of five bathroom and furniture cleaning methods. Only the bathroom tasks were significantly different (*p* < 0.05).

In addition to postures, subjects were able to perform most bathroom- and furniture-cleaning tasks with the long-handled, extendable tool at slower trunk speeds. For example, Figure 5 and Figure 6 show that off-plane trunk velocities were, in many cases, nearly double when the standard method was used. All cleaning tasks using the study tools produced much lower lateral velocities when compared to the standard method, on average (Figure 5), with all differences statistically significant. For transverse trunk velocities, only the bathroom-cleaning tasks generated lower average trunk-twisting velocities, with all differences statistically significant (Figure 6). As with posture, speed of work has long been associated with spinal loading and back injuries. Marras et al. [36] found spinal loads (assessed using muscle electromyography) to be significantly higher when trunk motions were dynamic compared to those measured when the trunk was static. Further, in a study by Bigos et al. [37], awkward working postures and the speed with which jobs were performed together accounted for 29% of high-cost back injury claims. Messing et al. [38] observed substantial time demands on female passenger train cleaners while cleaning toilets; in addition, the cleaners sustained a crouching position for a quarter of the time while performing this task. In a study of low back pain occurring to hotel housekeepers, Yusof et al. [39] found high Rapid Entire Body Assessment (REBA) scores for toilet-scrubbing indicating this bathroom-cleaning task is a high risk for MSD injuries.

These trunk kinematic results may actually under-represent the actual torso movements required by cleaners during their normal activities. Cleaners may be under time pressures during their actual work shifts, which can result in faster movements. Subjects in this study were permitted to take breaks, as needed, to reduce the potential for fatigue. The protocol used here was designed to allow for comparisons between study conditions not influenced by a subject’s physical condition.

**Figure 7:**
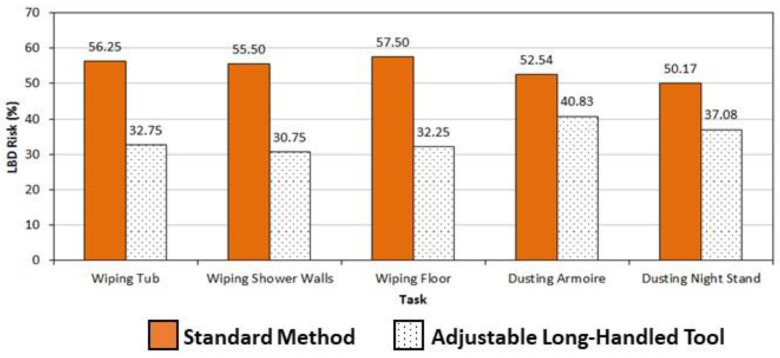
Differences in average LBD risk between cleaning methods of five bathroom and furniture cleaning methods. All were significantly different (*p* < 0.05).

This study has also demonstrated the impact that adjustable, long-handled tool use can have on reducing the degree of injury risk associated with hotel room cleaning work itself. The three trunk kinematic measures shown in Figure 4, Figure 5 and Figure 6 are also those used in the computation of LBD risk, a model determining the probability that an industrial job would have work exposures similar to those found to produce high injury rates of the low back [33]. These trunk measures, in combination with the frequency with which items (e.g., rags, tools) are handled during room cleaning and the external load these items place on the spine when being handled, produced LBD risk values that were significantly lower when adjustable, long-handled tools were used for all study tasks. Comparisons with standard cleaning methods are graphically presented in Figure 7. For bathroom-cleaning tasks, the standard method produced LBD risk values of 55% or higher, while use of the tool resulted in values of 33% or lower. For furniture-dusting, the difference between the methods was less, yet still significant: 50% or higher when using a rag versus 41% or lower using the long-handled, extendable duster. Putting these values in context, Marras et al. reported in earlier research (2000, personal communication), that the profiles of jobs with LBD risk values of 30% or less were most similar to those previously found to produce no low back disorders, while those above 60% were comparable to jobs having high rates of injuries to the low back. Thus, these results show that use of adjustable, long-handled tools, especially for bathroom-cleaning tasks, have the potential to reduce some hotel room cleaning tasks to a much safer level.

The finding that adjustable, long-handled tools reduced trunk motions during guest room cleaning is not surprising. These tools essentially serve to extend the arms and hands. As a result, the room cleaners were able to keep their backs more upright and use less-deviated off-plane motions when cleaning bathroom and furniture surfaces, while still being able to reach the needed surfaces. These results also serve as a complement to previously published work [21] on the impact of adjustable, long-handled tools. Kumar et al. [21] found lower perceived exertions scores among subjects when using an adjustable-handled tool to clean staircases, particularly because it allowed the task to be done using a less-abducted arm posture. Additionally, the cleaners reported the adjustable tool was comfortable to use. Öhrling and colleagues [40] found that cleaners reported use of an adjustable mop handle to be markedly more comfortable than one that could not be adjusted. In addition, most cleaners found the extendable tool to be easy to adjust, and the rest expressed the likelihood of using it after being provided time to practice. These are important considerations when addressing user adoption of new equipment and suggests that hotel room cleaners would be willing to integrate use of extendable-handle tools to perform their jobs. Such input from cleaners and the ability to train on cleaning equipment was recommended in a review article by Woods and Buckle [10] for improving musculoskeletal health among this workforce. While Kumar and Kumar [41] highlighted challenges to the adoption of comprehensive ergonomic interventions by employers, they concluded that tool design was a low-cost remedy likely to be put into practice. Although physical demands on the shoulder were not recorded for the present study, it was regularly observed that subjects were able to perform bathroom-cleaning and dusting tasks with their arms much less deviated (see Figure 2 and Figure 3 as examples). Therefore, it is reasonable to assume that these tools may also reduce demands on both the shoulder as well. This finding indicates that the modification does not create new concerns, which is an-other important consideration when introducing new tools or work practices for a task. Furthermore, this indicates the potential impact of study tools to reduce MSD injuries in most areas that were the leading individual parts of body affected in hotel room cleaners in the Buchanan study—back and shoulders [12].

### 4.2. Significance of Findings to Provide Ergonomic Interventions for Hotel Room Cleaner Tasks

To the study authors’ best knowledge, this is the only study of adjustable, long-handled cleaning tools used by hotel room cleaners to perform the key tasks of bathroom-cleaning and furniture-dusting. The significant reduction in all study metrics using the risk-reduction tools contributes to the necessity for ergonomic interventions for this occupation. The call for such interventions has been most recently underscored for improving hotel room cleaners’ health [15] and to reduce bending, awkward postures and reaching that contribute to musculoskeletal injury risk [42] and cardiovascular demands [24].

This study demonstrates that adjustable, long-handled tools are a solution to reduce risk factors for musculoskeletal injury risk, in particular low-back disorders and possibly other affected body parts. Furthermore, these tools are a viable option for hotel employers to provide to their workforce where workplace evaluations and injury investigations indicate the need for solutions. A recent review of workplace evaluations performed as part of compliance with the Cal/OSHA Hotel Housekeeping Musculoskeletal Injury Prevention standard [43], promulgated in 2018, found a lack of implementation of recommendations for tools and equipment. Out of 54 California hotels studied for 2018 through 2020, only one property obtained extendable cleaning tools for bathroom-cleaning tasks [42]. With hotel room cleaners accounting for the largest proportion of a hotel’s workforce, adopting the use of adjustable, long-handled tools as an injury risk-reduction method for this predominant hotel occupation and its leading cleaning tasks needs to be prioritized.

### 4.3. Limitations

There are some limitations to this study that should be addressed. First, only the trunk motions required of these tasks were quantified. A more-comprehensive study would have assessed demands on the other body parts (e.g., shoulders, hands/wrists) where hotel room cleaners have reported injuries [12], as well as other known influences. For example, comprehensive literature reviews of MSD causal pathways, such as by the National Research Council [44], have concluded that workplace psychosocial factors (e.g., work pace, stress from work demands, job satisfaction) are related to LBD risk and that individual traits, such as age and gender, can mediate the impact of work on the spine. It was beyond the scope of this effort to conduct such a broad assessment of hotel cleaner injury risk exposures, and no metric is known to exist that quantifies the collective impact of known MSD causes. In addition, it should be reemphasized that LBD risk model associated with LMM and other work demand data has been validated [34].

Second, the study size is determined based on possible variability in dependent measures; this can include work experience. It was not our intent in this study to examine trunk kinematics variability due to experience. Even if any inexperienced cleaner(s) had vastly different trunk motions, the overall kinematic results still produced large, significant differences between cleaning methods.

Third, task time differences due to the cleaning equipment used was not studied. However, Janowitz et al. [20] found there to be no significant time difference between use of a sponge and a handled tool to clean a standard American shower/tub area. We expect that similar results would have been found in this study as well.

Fourth, the study occurred within only one type of hotel guest room. Guest room designs can vary within a hotel (i.e., tub vs. walk-in shower, sizes and shapes of armoires and nightstands, etc.), and considerably between hotel chains and between brands within the same hotel chains, e.g., from luxury to extended-stay hotels. The standard room design chosen for this study was typical of those found throughout the U.S. in the category of mid-priced hotels. Although the actual kinematic data may have differed had another room type been chosen, this study focused on comparisons between alternative work practices in the same type of guest room. Thus, we believe similar results would have been found had this study been conducted in another hotel having a different room configuration or price point but by comparing alternative work practices within the same type of guest room.

## 5. Conclusions

This study addressed several ergonomic issues of concern for hotel room cleaners, whose jobs require a wide variety of physical efforts. It demonstrated that tools with adjustable, long-handles available in the general marketplace can be used in the hospitality industry as an injury risk reduction method when performing key guest room-cleaning tasks, resulting in decreased low back disorder risk. Because reaching and bending are required for many other room-cleaning tasks not studied here (e.g., washing windows, dusting baseboards), these results suggest that similar tools used for these purposes may further improve the health and safety of individuals who do this work for a living. This study contributes toward filling the gap in the literature about ergonomic solutions to occupational injury hazards found in cleaning tasks common to hotel room cleaners, namely bathroom-cleaning and furniture-dusting. It also adds to the literature that has found that adjustable, long-handed tools are a safe alternative to close-up cleaning methods that present multiple known hazards, such as low back disorders and slips, trips, and falls. It is reasonable to extrapolate that adjustable, long-handled tools can increase worker safety by eliminating the need for cleaners to climb into tubs or onto tub edges to clean shower walls and hard-to-reach tub areas or onto vanities to clean large mirrors. This study demonstrates the importance of both assessing the LBD risk of specific hotel room cleaners’ job tasks and considering the decisions to select appropriate tools and equipment as part of an injury risk-reduction program. These findings indicate the potential to reduce MSD injuries to the low back and other body parts of hotel room cleaners. Moreover, one can reasonably infer that these findings may apply to cleaning and housekeeping work in other industries, such as healthcare, building services and nursing and elder care facilities, where bathroom-cleaning and furniture-dusting tasks are performed. Future studies using the study tools could prove beneficial to these other sectors where similar cleaning tasks are performed.

## Figures and Tables

**Table 1:** Means and standard deviations (SD) for trunk kinematic and LBD Risk measures, for three hotel bathroom-cleaning tasks. Range of motion values are in degrees, velocities are in deg/s, and accelerations in deg/s^2^.

Trunk Kinematic Measure	Wiping Tub				Wiping Shower Walls				Wiping Floor			
	Standard Method		Adjustable Long-Handled Tool		Standard Method		Adjustable, Long-Handled Tool		Standard Method		Adjustable, Long-Handled Tool	
	Mean	*SD*	Mean	*SD*	Mean	*SD*	Mean	*SD*	Mean	*SD*	Mean	*SD*
Lateral Plane												
Max. ROM ⁑	10.66	*4.26*	6.39 *	*1.94*	18.97	*4.44*	9.81 *	*3.30*	14.19	*4.49*	6.47 *	*2.01*
Avg. Velocity	5.29	*1.69*	3.01 *	*1.04*	7.59	*1.64*	3.93 *	*1.61*	6.08	*1.38*	3.19 *	*0.72*
Max. Velocity	21.78	*6.84*	11.69 *	*4.33*	25.77	*5.14*	14.06 *	*4.96*	24.62	*5.99*	12.55 *	*3.96*
Max. Acceleration	151.82	*53.41*	81.15 *	*31.98*	159.58	*38.11*	84.31 *	*28.05*	150.69	*44.25*	80.36 *	*23.88*
Sagittal Plane												
Max. Flexion	24.02	*8.88*	1.56 *	*5.89*	14.34	*10.24*	−1.56 *	*5.98*	26.93	*7.98*	7.44 *	*9.14*
Avg. Velocity	6.95	*1.35*	3.60 *	*0.99*	10.98	*2.59*	5.17 *	*1.74*	8.55	*2.41*	4.52 *	*1.89*
Max. Velocity	24.84	*7.93*	13.74 *	*2.88*	37.44	*9.18*	18.54 *	*4.96*	31.94	*11.90*	17.00 *	*7.60*
Max. Acceleration	154.39	*60.34*	89.43 *	*16.92*	225.49	*59.34*	118.97 *	*28.06*	197.85	*96.51*	106.27 *	*39.99*
Transverse Plane												
Max. ROM ⁑	9.89	*4.98*	4.85 *	*2.54*	11.25	*2.36*	8.07 *	*2.68*	9.12	*3.45*	4.83 *	*1.65*
Avg. Velocity	4.90	*2.83*	1.95 *	*1.25*	5.83	*1.62*	3.25 *	*1.24*	4.13	*1.50*	2.03 *	*0.71*
Max. Velocity	23.18	*9.22*	12.81 *	*4.85*	26.45	*6.72*	18.51 *	*6.80*	20.72	*5.81*	12.52 *	*2.93*
Max. Acceleration	166.57	*65.26*	96.55 *	*35.28*	195.16	*49.07*	139.21 *	*49.73*	145.04	*37.76*	97.61 *	*24.31*
LBD Risk (%)	56.25	*15.66*	32.75 *	*22.00*	55.50	*8.39*	30.75 *	*6.92*	57.50	*14.02*	32.25 *	*6.97*

* Denotes statistical significance at α = 0.05. ⁑ Range of Motion.

**Table 2:** Means and standard deviations (SD) for trunk kinematic and LBD Risk measures, for two hotel guest room dusting tasks. Range of motion values are in degrees, velocities are in deg/s, and accelerations in deg/s^2^.

Trunk Kinematic Measure	Dusting Armoire				Dusting Nightstand			
	Standard Method		Adjustable, Long-Handled Tool		Standard Method		Adjustable, Long-Handled Tool	
	Mean	*SD*	Mean	*SD*	Mean	*SD*	Mean	*SD*
Lateral Plane								
Max. ⁑ ROM	16.70	*5.38*	10.50 *	*3.80*	11.79	*5.24*	8.16 *	*2.67*
Avg. Velocity	7.13	*1.94*	5.12 *	*1.34*	6.58	*2.37*	4.79 *	*0.94*
Max. Velocity	24.88	*6.24*	18.44 *	*4.59*	21.89	*7.35*	15.56 *	*3.49*
Max. Acceleration	154.03	*42.05*	115.88 *	*32.81*	144.76	*59.22*	100.67 *	*30.37*
Sagittal Plane								
Max. Flexion	23.04	*10.93*	6.96 *	*10.01*	23.86	*9.24*	11.12 *	*9.88*
Avg. Velocity	11.93	*4.00*	7.97 *	*3.67*	8.93	*2.09*	5.65 *	*2.47*
Max. Velocity	40.14	*10.75*	26.05 *	*11.31*	27.93	*6.30*	18.50 *	*8.17*
Max. Acceleration	237.17	*83.09*	153.00 *	*66.03*	173.16	*48.52*	118.15 *	*46.17*
Transverse Plane								
Max. ⁑ ROM	8.33	*1.58*	7.55	*2.01*	5.19	*1.96*	4.24	*1.88*
Avg. Velocity	4.26	*1.57*	4.37	*1.42*	3.46	*1.62*	2.38	*0.99*
Max. Velocity	20.43	*4.76*	20.01	*4.41*	14.58	*5.36*	13.19	*4.95*
Max. Acceleration	158.51	*38.24*	149.16	*38.30*	113.61	*34.18*	94.68	*30.27*
LBD Risk (%)	52.42	*6.04*	40.83 *	*10.43*	50.17	*6.09*	37.08 *	*9.35*

* Denotes statistical significance at α = 0.05. ⁑ Range of Motion.

## Data Availability

All data gathered for this project is contained in a secure location at The Ohio State University and is not available for public access.
